# Time-Resolved Spectroscopy of Ethanol Evaporation on Free-Standing Porous Silicon Photonic Microcavities

**DOI:** 10.3390/ma11060894

**Published:** 2018-05-26

**Authors:** María del Rayo Jiménez Vivanco, Godofredo García, Rafael Doti, Jocelyn Faubert, Jesus Eduardo Lugo Arce

**Affiliations:** 1 Centro de Investigación en Dispositivos Semiconductores, Instituto de Ciencias (ICUAP), Benemerita Universidad Autonoma de Puebla (BUAP), Ciudad Universitaria, Puebla, Pue. C.P. 72570, Mexico; jimenezvmr10@gmail.com (M.d.R.J.V.); godgarcia@yahoo.com (G.G.); 2 Faubert Lab, School of Optometry, University of Montreal, Montreal H3T 1P1, QC, Canada; rafael.doti@gmail.com (R.D.); jocelyn.faubert@gmail.com (J.F.)

**Keywords:** porous silicon, ethanol evaporation, time window, shift rates

## Abstract

In this work, we have followed ethanol evaporation at two different concentrations using a fiber optic spectrometer and a screen capture application with a resolving capacity of 10 ms. The transmission spectra are measured in the visible-near-infrared range with a resolution of 0.5 nm. Porous Silicon microcavities were fabricated by electrochemistry etching of crystalline silicon. The microcavities were designed to have a localized mode at 472 nm (blue band). Ethanol infiltration produces a redshift of approximately 17 nm. After a few minutes, a phase change from liquid to vapor occurs and the localized wavelength shifts back to the blue band. This process happens in a time window of only 60 ms. Our results indicate a difference between two distinct ethanol concentrations (70% and 35%). For the lower ethanol concentration, the blue shift rate process is slower in the first 30 ms and then it equals the high ethanol concentration blue shift rate. We have repeated the same process, but in an extended mode (750 nm), and have obtained similar results. Our results show that these photonic structures and with the spectroscopic technique used here can be implemented as a sensor with sufficient sensitivity and selectivity. Finally, since the photonic structure is a membrane, it can also be used as a transducer. For instance, by placing this photonic structure on top of a fast photodetector whose photo-response lies within the same bandwidth, the optical response can be transferred to an electrical signal.

## 1. Introduction

Porous silicon (Psi) has been used for different applications due to their low cost and uncomplicated fabrication process, usually by electrochemical etching. Thanks to the modulation of the porosity, refractive indexes can be modulated from 3.6 to 1.1. This modulation is achieved by the application of different current pulses. This technique allows for us to design multilayer structures that are based on porous silicon, like distributed Bragg reflectors (DBRs) [1,2,3], rugate filters [3,4], and microcavities (MC) [5,6,7]. Furthermore, due to the high internal surface area of Psi, all of the devices mentioned above are good candidates for sensing biological and chemical substances [8,9,10] and gas [11,12,13]. The microcavities can be obtained for different wavelengths within the Vis-IR range on a silicon substrate. In several studies, they have been exposed to various solvents, such as different types of alcohols, including wines [14], ethylene glycol [15], deionized water and glucose solutions [16], and other solvents [6]. The adsorption of these types of molecules inside the pores changes the optical characteristics of the MC. MCs can be designed to have a localized mode inside its photonic band gap whereby the light can be trapped within the MC. The presence of the solvents that were mentioned before usually induces a red shift in the localized energy mode. That is the localized mode frequency peak shifts towards the low energy range. This operation is known as a typical sensing operation; the explanation of this shift is primarily due to the increase in the refractive index value within the microcavity by filling the pores with solvents that have a higher refractive index value and replace the air inside the pores.

Another recent sensing technique for vapor uses Bloch surface waves (BSW), excited by attenuated total reflection (ATR) showing that the vast Psi surface area that is associated with the extremely narrow BSW resonances lead to enhanced sensitivity. In the same research, it is also reported that when the vapor is completely pumped out, and the flow cell is again filled with the environment air, the BSWR shifts back to its original position. This sensor was based on freestanding Psi membranes [17]. In other studies, it was demonstrated that the microcavities have a blue shift when they are immersed in 1 mM HAuCl4 solution of ethanol at different times. The blueshift was strongly correlated with Au deposition time resulting in the change of the effective refractive index on the microcavities surface. A simulation also showed how the MCs reflectance spectrum changes to verify the detection mechanisms as an optical sensor [18]. Psi rugate filters have been reported for alcohol and toluene sensing in the vapor phase. They were chemically patterned with hydrophilic (3-aminopropyltriethoxysilane, APTES) and hydrophobic (pentafluorophenyl dimethylchlorosilane, PFPS) silane compounds. After this chemical patterning procedure, the rugate filter showed two reflectance peaks at 623 nm and 795 nm for the PFPS and the APTES. The position of the reflectance peak on the hydrophilic region remained constant and it acted as an internal reference to the shift of the hydrophobic reflectance peak. The separation between these two peaks was 172 nm. When the rugate filter was exposed to alcohol and toluene, the PFPS undergoes a redshift towards wavelengths that were closer to the APTES peak. Also, the rugate filters were exposed to different alcohol vapor concentrations; as the vapor concentrations increased, the peak separation between the PFPS and APTES decreased [19].

In the present work, we show sensing data of alcohol dilution at two different concentrations using distilled water on the membrane of a microcavity that was manufactured with porous silicon (freestanding Psi) in the near-infrared and the visible optical range. We mainly follow a localized mode (472 nm) and an extended mode (750 nm) of the microcavity, and observed that both of the modes present a redshift first when liquid ethanol is infiltrated, and then after ethanol evaporation begins, the shift direction is reverted. These changes were monitored in real-time with a resolution of 10 ms. Using this technique, we observed the phase change of ethanol from liquid to vapor. Our results show that these photonic structures plus the spectroscopic technique can be used as a sensor with enough sensitivity and selectivity. Moreover, by enhancing the time resolution of this technique, the evaporation kinetics could also be known. Finally, since the photonic structure is a membrane, it can easily be adapted to some other optical or electrical devices to create a transducer.

## 2. Materials and Methods

### 2.1. Porous Silicon Microcavity Fabrication

The porous silicon was obtained by the electrochemical anodization of crystalline silicon wafers (c-Si) *p*+ type doped with boron (100) orientation with resistivity (0.01–0.02 Ω-cm), in an aqueous electrolyte of 40% HF and ethanol 99.7% at room temperature with a volume ratio of 1:1. Electrochemical anodization is performed in a Teflon cell; a tungsten electrode immersed in the electrolyte was used as the cathode, an aluminum plate that contacts the unpolished backside of the c-Si wafer was used as the anode, and the etching area in the wafer was 1 cm^2^. Before anodization, the p-type Si wafer was immersed in a solution of 20% HF for 5 min in order to remove native oxide.

The microcavity consists of a defect with a localized mode between two DBRs. They are obtained by alternating layers of a high refractive index (*H*) and low refractive index (*L*), fulfilling the Bragg condition, $n_{i}d_{i}=\frac{\lambda }{4}$, where i=H,L. The defect must meet the condition $n_{i}d_{i}=\frac{\lambda }{2}$, and the MC has the following sequence:

$${\left(HL\right)}_{X8}L\;{\left(HL\right)}_{X7}$$

In the experimental procedure, a current pulse of 5 mA/cm^2^ (low porosity) is applied for 4.1 seconds (s). The second step consists in using a current pulse of 80 mA/cm^2^ (high porosities) for 1.1 s; the defect is obtained by using a current pulse of 80 mA/cm^2^ for 2.2 s. After each current pulse, a pause of 3 s is introduced in order to generate the flow of the electrolyte and to prevent porosity gradients. The microcavity was lifted off from the c-Si substrate applying a high current pulse of 450 mA/cm^2^ for 2 s, and it was placed on a quartz substrate and then dried in the environment. The current profile of the MC was delivered by a power supply (Keithley 2460, Aurora Road, Cleveland, UT, USA controlled by a computer. The porosities that we measured for the MCs were 39% and 74%, and the thickness of each layer is $d_{H}=30.2\;\mathrm{nm}$ and $d_{L}=64.6\;\mathrm{nm}$. These parameters were obtained by gravimetric measurements. High resolution scanning electron microscopy JEOLJSM7600F (JEOL, Peabody, MA, USA) was used to visually ensure the presence of the multilayers and the defect regions in the MC.

### 2.2. Porous Silicon Refractive Index

Through the electrochemical anodization of c-Si wafers, Psi monolayers were obtained. The porosity and the thickness for each monolayer of Psi are measured using gravimetric methods. To get the refractive index of each Psi monolayer, we used the effective medium theory of Bruggeman, while considering that we have a homogeneous medium that is associated with an effective dielectric function [20]. This effective dielectric function is related to the dielectric functions of the two mediums forming this material (air and silicon), where it is assumed that all of the pores or islands of the bulk material experience an average electric field [21]; the equation is expressed as follows:

(1) $$p\frac{\varepsilon_{air}-\varepsilon_{Psi}}{\varepsilon_{air}+2\varepsilon_{Psi}}+f\frac{\varepsilon_{Si}-\varepsilon_{Psi}}{\varepsilon_{Si}+\varepsilon_{Psi}}.$$

where p is the volume fraction of air within the Psi layer (porosity); $\varepsilon_{air}$ is the dielectric function of air; $\varepsilon_{Si}$ is the dielectric function of c-Si, and $\varepsilon_{Psi}$ represents the dielectric function of Psi; and, f=1−p is the volume fraction of c-Si in the porous layer.

The dielectric function is a complex number, and it is related to the complex refractive index, which is composed of real and imaginary parts. Its real part represents the ordinary refractive index when no absorption of light is taking place. The imaginary part is known as the extinction coefficient. The extinction coefficient determines the absorption rate in the medium [22]. The porosity can be estimated then by using the Bruggeman approximation, providing that we know the dielectric functions of each component (air and c-Si).

### 2.3. Porous Silicon Microcavity Ethanol Sensing

Ethanol sensing was performed, as follows: First, we measured the transmission spectrum of the MC to have a reference. Second, we submerged the microcavity in 5 mL of ethanol 70% by 1 min. The MC was exposed to liquid ethanol without immersing the quartz substrate completely. Once the MC gets impregnated with liquid ethanol, the MC is placed on the fiber optic spectrometer (Stellarnet, Tampa, FL, USA) that is set up to measure optical transmission. The transmission spectrum from 350 nm to 900 nm was recorded every 10 ms using a screen capture application. The immersion of the MC on liquid ethanol redshifted the whole transmission spectrum. The transmission spectra returned to the reference point after of 60 ms. After recording the dynamics of the entire bandwidth of wavelengths to a point where no noticeable changes were observed, the microcavity was removed and dried up for 5 min in order to begin a new sensing process. We repeated this procedure three times. The same protocol was used in a second experiment where we submerged the microcavity in a solution 1:1 of ethanol and distilled water. The measurements were taken at 22 °C with a pressure of 761.311 mmHg and 31% of humidity.

## 3. Results

### 3.1. Porous Silicon Microcavity Fabrication

**Figure 1:**
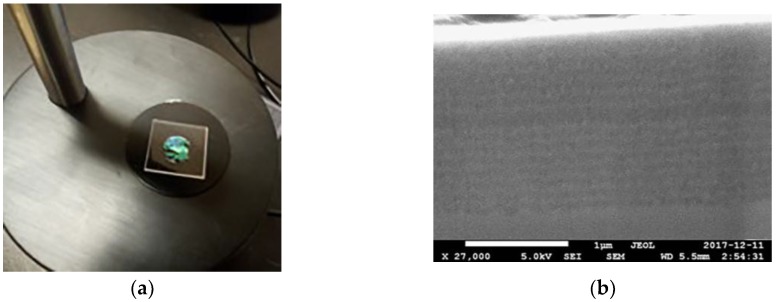
These figures show the membrane of a microcavity on a quartz substrate and an SEM measurement of a microcavity deposited on $p^{+}$ silicon substrate. (**a**) The membrane of a microcavity on a quartz substrate that is used for the sensing measurements; and (**b**) Cross-section SEM of a microcavity on $p^{+}$ silicon substrate with 31 layers. The existence of a microcavity and the contrast between both of the refractive indexes are shown. The membrane has an area of 1 cm^2^.

Figure 1a shows the free-standing membrane microcavity. It has a surface of 1 cm^2^, and it was self-supported on a quartz substrate with dimensions of 1.6 cm × 1.6 cm. Figure 1b shows a high-resolution scanning electron microscopy image of the microcavity on a $p^{+}$ silicon substrate. The layers with low porosities are in light gray, and the layers with high porosities are in dark gray. The presence of a spatial defect between two DBRs can be seen. The porosities that we measured for these MCs were 39% and 74%, and the thickness of each layer is $d_{H}=30.2\;\mathrm{nm}$ and $d_{L}=64.6\;\mathrm{nm}$. These parameters were obtained by gravimetric measurements. According to theoretical calculations, the microcavity has a total depth of 1.5 μm, while SEM measurements show an overall depth of approximately 1.6 μm with thicknesses of $d_{H}=35\;\mathrm{nm}$ and $d_{L}=68.75\;\mathrm{nm}$. These values are of the same order of magnitude as the ones that were found with the gravimetric method with a difference of approximately 4.8 nm in each layer. The SEM values were used in the theoretical calculations.

### 3.2. Porous Silicon Refractive Index

Consider the experimental and the theoretical values that were obtained for porosity and thicknesses shown in Table 1. For the experimental values, porosity was measured by gravimetric methods, and its value is utilized to calculate the refractive index using the Bruggeman formula. For the theoretical values, we used the refractive indexes as free parameters to fit the experimental transmission spectra (Section 3.3). Once a good fit is achieved, we used those refractive indexes values and Bruggeman’s equation in order to determine the porosity.

### 3.3. Porous Silicon Microcavity Bandgap Structure

**Figure 2:**
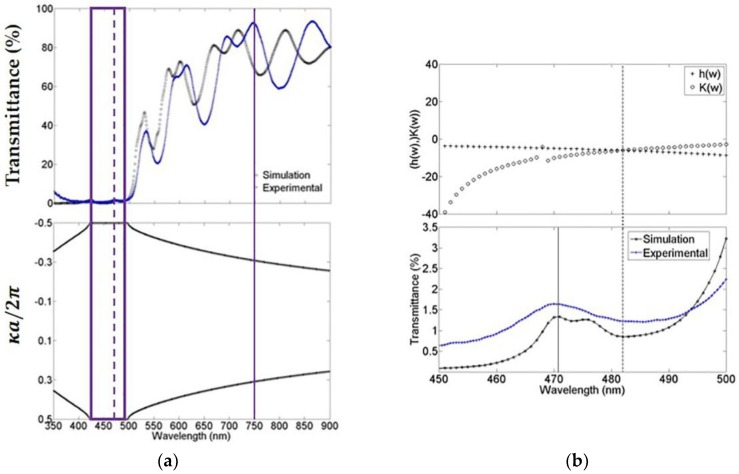
These figures show a comparison of the photonic band structure and localized mode calculation. (**a**) The theoretical and experimental transmission spectrum of a microcavity that was obtained in the visible-near-infrared range, its localized mode at 472 nm (broken line) and its bandgap (empty box); and (**b**) Comparison of both theoretical localized mode values and the experimental value that was measured with its spectrum of transmission. For the bandgap (a, bottom) and localized mode calculation (b, top), we used the mean of the complex refractive indexes norm within the whole wavelength range being explored.

The experimental photonic bandgap structure was determined by measuring the transmittance spectrum of MCs using a Stellarnet fiber optical spectrometer with a wavelength resolution of 0.5 nm. Figure 2a (top) shows the theoretical and experimental comparison of the microcavity transmission spectrum. In general, the fit is good, and in both cases, we observed that the absorption of porous silicon at short wavelengths is powerful. Nevertheless, even with this light absorption, the microcavity is capable of sustaining a localized mode at 472 nm (broken line) and an extended mode at 750 nm (solid line). Figure 2a (bottom) the theoretical bandgap structure of the microcavity is presented. We can observe the existence of a forbidden bandgap within the first Brillouin zone where the light cannot propagate. The experimental and theoretical forbidden photonic bandgap edges are 423 nm and 498 nm (empty box). Experimentally, the localized mode is found between these edges at exactly 472 nm. Figure 2b (top) shows the region from 450 nm up to 500 nm, approximately where the forbidden photonic bandgap was found. The theoretical localized mode is located at 482 nm, and it is calculated by using a combination of the variational method and the transfer matrix method (see Supplementary Materials). Figure 2b (bottom) shows the microcavity theoretical and the experimental transmission spectrum that were obtained before. The spectra were plotted in the range from 450 nm up to 500 nm. In this plot, we can distinguish the position of the theoretical localized mode calculated with the combinational method, as described in Figure 2b (Top, broken line) [23] and it is compared with the result that was obtained using the transference matrix method (solid line) [24]. The latter method predicts the localized mode position with a difference of less than 2 nm when compared with the experimental position, while the former method prognosticates the localized mode with a difference of 10 nanometers from the position of the experimental localized mode.

### 3.4. Porous Silicon Microcavity Ethanol Sensing

**Figure 3:**
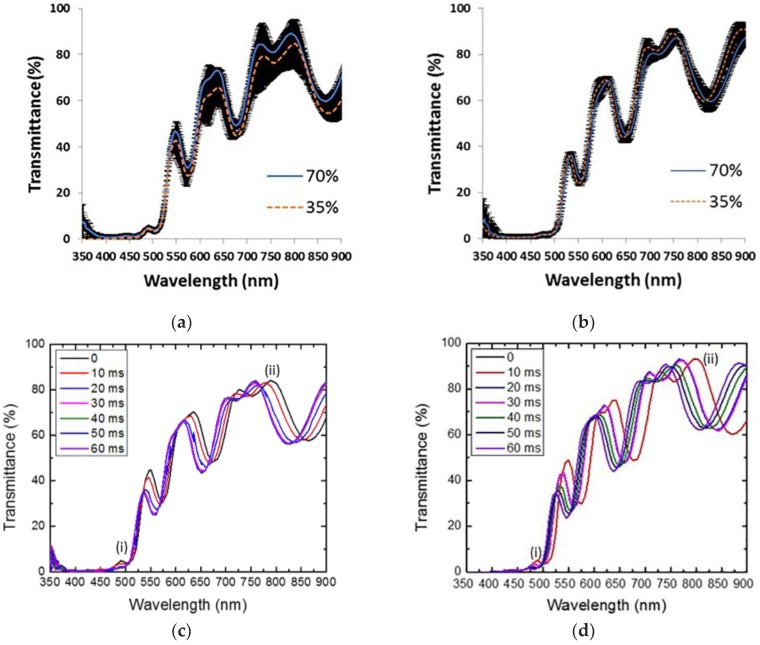
Sensing measurements result for two different ethanol concentrations (70% and 35%). (**a**) Average transmission spectra (three trials for each concentration) at 0 ms. (**b**) Average transmission spectra (three trials for each concentration) at 60 ms. The black band represents a one-standard error. In both cases, the average lies within the error band, meaning that, on average, both mode peaks begin and end at the same wavelength position. (**c**) Example of one trial showing the wavelength shift towards low energy with a concentration of alcohol of 70%; (**d**) The same as (**c**) with a concentration of alcohol of 35%.

We have followed ethanol evaporation employing a fiber optical spectrometer and a screen captures application with a 10 ms resolution, the measurements were taken from 350 nm to 900 nm. Figure 3 shows the sensing measurements results for two different ethanol concentrations at 70% and 35%. We have averaged the three trials for each ethanol concentration. The average for each concentration is compared in two separated panels. (a) This panel belongs to 0 ms (liquid ethanol) and (b) for 60 ms (ethanol depleted). We can see that the averages at the beginning and 60 ms are statistically the same. The standard error (marked by the black band) engulfs both of the averages. We can hypothesize that the initial and final position of the membrane caused by mechanical stress, during both liquid infiltration and ethanol desorption is the main reason for the fluctuations on the transmission amplitude and wavelength position, which are displayed in Figure 3a,b.

Figure 3c shows the result of sensing alcohol at 70%, from 0 ms up to 60 ms (milliseconds). One can observe a redshift of approximately 17 nm and 43 nm for localized (i) and extended (ii) modes, respectively, initially being located at 472 nm and 750 nm. Once the mode peaks are shifted from their initial positions; after a few seconds, both of the modes come back near to their initial positions and recover their initial values.

In Figure 3d, we present the sensing measurements for alcohol at 35% with the same time window. The shift of the transmission spectrum of the microcavity towards long wavelengths is also presented. In both cases, the localized mode and the extended mode are shifted first (liquid ethanol infiltration) towards long wavelengths and then towards short wavelengths (ethanol evaporation).

**Figure 4:**
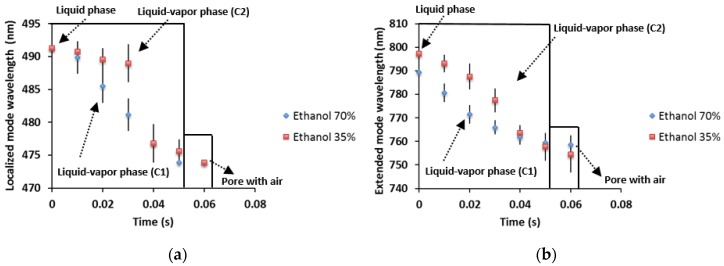
Comparison of liquid ethanol evaporation with concentrations of 70% and 35% in a localized mode and an extended mode as a function of time. (**a**) This figure shows the shift of the localized mode as a function of time by the ethanol evaporation for concentrations of 70% and 35%. (**b**) The shift of the extended mode towards its initial position as a function of the time by the phase change from liquid to vapor of ethanol at 70% and 35% concentrations.

In Figure 4a, we present the sensing measurements of time-resolved spectroscopy of ethanol evaporation at 70% and 35% concentrations. It is shown that the phase change takes place during 60 ms. The first stage is the liquid phase, where the pores are full of liquid ethanol, and a redshift of the localized mode is found. After that ethanol evaporation begins and the localized mode wavelength changes location and blueshifts. The acronym C1 represents the liquid-vapor phase with ethanol at 70%; the evaporation has a more linear behavior with time than in the case of ethanol at 35% (acronym C2), which has a nonlinear response. When ethanol evaporation is finished, the pores are full of air again, so the localized mode shifts back to the blue band in both cases. The comparison of the kinetics of ethanol evaporation for the extended mode is shown in Figure 4b, as before this has a more linear behavior for ethanol at 70% (acronym C1) and a nonlinear behavior for ethanol at 35% (acronym C2). We may have three elements that could influence the non-linear response of 35% ethanol. One can be the humid air from the atmosphere. Since the experiment is performed in the laboratory with an open atmosphere so that the alcohol evaporation induces a local temperature drop (in the substrate) that can originate water (from the air) condensation. Another critical parameter is the high volumetric surface density of porous silicon that, when combined with the phase change, it could enhance the refrigerator effect and finally the local appearance of Ethanol-Water Azeotrope (nonlinear surface tension). The main differences between both of the ethanol concentration results are that the evaporation process of ethanol at 70% happens in 60 ms. While for lower ethanol concentration, the blue shift rate process is slower for the first 30 ms and then equals the high ethanol concentration blue shift rate. This result means that in this particular case, the extra water added is evaporated along with ethanol within a 30 ms window. In the case of the extended mode, the wavelength shift is higher by approximately 43 nm, because in this range, a small change in the refractive index makes a big difference in the shift.

Vapor pressure differences between water and ethanol take place in the kinetics of evaporation, as shown in Figure 4a,b. The behavior of desorption process is described by a desorption isotherm, where the influence of the vapor pressure in the desorption rate is noticeable. The behavior that is observed in Figure 4a,b shows a loss of the linearity at the end of the process; this is characteristic of adsorption and desorption isotherms.

**Figure 5:**
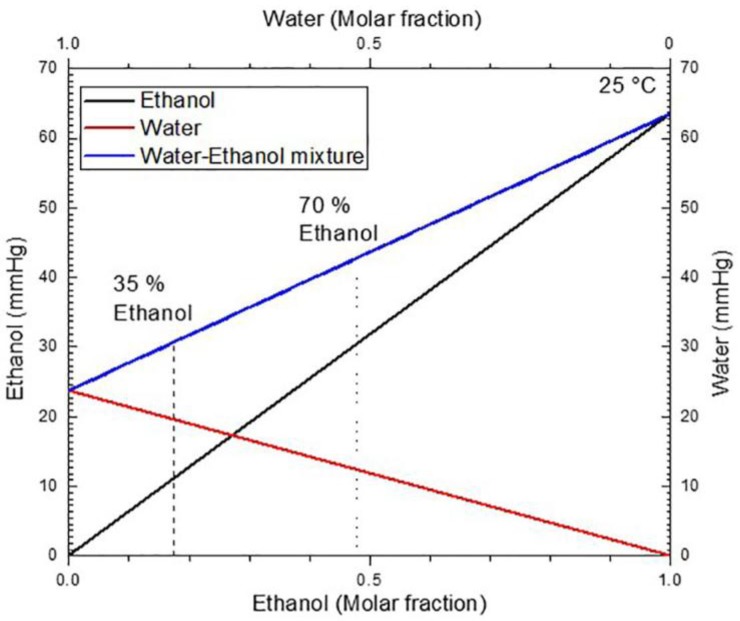
Vapor pressures as a function of the molar fraction for ethanol (black line), water (red line), and the mixture (blue line) at 25 C. Vapor pressure of the mixture for ethanol concentrations in a volume of 35% and 70% are indicated (dot lines).

The vapor pressure of the water-ethanol mixture can be seen in Figure 5, this diagram was obtained using Raoult’s law. The plot shows that an increase of the molar fraction for ethanol in the mixture increases the vapor pressure. Therefore, it is natural that the desorption rate increases for the mix of ethanol at 70%. This desorption increase can be seen in Figure 4a,b (acronym C1), where ethanol evaporation is fast when compared to the 35% ethanol concentration (acronym C2). In the latter case, the desorption is more remarkable in the first 30 ms than in the last 30 ms.

## 4. Discussion and Conclusions

We designed and fabricated a freestanding membrane microcavity that was based on porous silicon. The photonic band structure was measured employing the transmission spectrum and theoretically using the matrix transfer method, and the agreement was reasonable. From the transmission spectrum, one can observe the photonic bandgap and possibly a localized state. To be sure that this is true, we calculated the MC photonic bandgap and the ideal location of the localized state. We obtained a difference of 10 nm concerning the experimental value of 472 nm. The agreement between theoretical and experimental bandgap intervals was good as well. This further theoretical analysis is always necessary in order to be sure that a localized mode has been efficiently created within the bandgap. Most of the sensing studies that use localized modes are only experimental or only calculate the transmission spectrum, which is not enough. Surface modes can also appear within the bandgap, and they look like sharp peaks as well in the transmission spectrum. One example of this kind of sensor is described in reference [17], which is also a porous silicon membrane. Nonetheless, the mechanism to excite the surface state requires the use of the well-known ATR technique, thus making the sensing mechanism more complicated. Another type of sensor is mentioned in reference [25], where a porous silicon microcavity is used but it was not lifted off from the silicon substrate. The sensor is used for sensing ethanol and acetone in their vapor phases with different concentrations. The wavelength shift for different levels of ethanol at the vapor phase was small when compared to our results. A study of alcohol and toluene sensing in the vapor phase on rugate filters has been presented. There, the authors showed that it is possible monitor the reflectance spectrum of two different regions, which were chemically patterned with APTES and PFPS, the result of the measurement showed two reflectance peak maxima at 623 nm and 795 nm for each rugate filter than correspond to PFPS and APTES. The position of the reflectance peak on the APTES region remained constant and it acted as an internal reference to the shift of the PFPS reflectance peak; the reflectance spectrum was monitored over time [19].

We presented a sensing method that allows for us to observe ethanol evaporation at different concentrations. The technique uses a fiber optical spectrometer with a wavelength resolution of 0.5 nm and an application that captures the computer screen at a sampling rate of 10 ms. Liquid ethanol infiltration produces a wavelength redshift in the whole transmission spectrum, followed by a blueshift after the evaporation of ethanol begins. We tracked down the shift of two particular wavelengths (localized and extended modes) that were located at 472 nm and 750 nm.

We have also demonstrated that the use of a localized state is not mandatory in this sensing technique. An extended state can do the job. The reason is the use of the fiber optic spectrometer that provides the whole transmission spectrum with a resolution of 0.5 nm. Current fiber optical spectrometers have become small and affordable, which opens up the possibility to integrate them along with MCs or other types of photonic structures.

Another critical point to make is that in almost all of the sensing devices based on MCs, the localized modes are within the near infrared region [3,4,5,7]. The reason is that Psi nanostructures do not absorb light at those wavelengths. However, by going into the visible region, light absorption begins to play an import role in smearing the bandwidth of the bandgap and the localized mode. This smearing is manifested in our experiments. Although we know in theory and by SEM, that we have achieved the creation of the localized mode, its amplitude and bandwidth are small. The reason of this is because the localized mode is in the blue band (472 nm), where light absorption cannot be neglected. However, this is enough to show our sensing scheme. We decided to experiment in this high light absorption region in order to demonstrate that it is possible to expand the optical working bandwidth of current MC based on Psi. In the future, we will enhance the optical features (amplitude and bandwidth) of the localized mode in the blue region and probably UV regions by carefully playing with the oxidation of the Psi nanostructures. Furthermore, since the photonic structure is a membrane, and with the objective of developing an electro-optic device in the future, we decided to study the transmission properties of this material, because this could be integrated as a filter combined with a photodetector, configuring a typical centered optical system. The lifespan of the material that was considered for this potential development (even within a few hours) is enough for a disposable (but precise) filter. It will be a low-cost device component that can be easily adapted to some other optical or electrical device in order to create a transducer at all visible and near-infrared wavelengths as well.

In conclusion, we presented the sensing measurements of time-resolved spectroscopy of ethanol evaporation at concentrations of 70% and 35%. It is shown that the phase change lasts around 60 ms. The first stage is the liquid phase, where the pores are full of liquid ethanol after that ethanol evaporation begins and the localized mode wavelength changes location due to the decrease of the refractive index value. We mainly follow a localized mode (472 nm) and an extended mode (750 nm) of the microcavity, and observed that both modes present a redshift first when liquid ethanol is infiltrated, and then after ethanol evaporation begins the shift direction reverts. Our results show that these photonic structures plus the spectroscopic technique can be used as a sensing technique with enough sensitivity and selectivity. Future work will be directed on enhancing the time resolution of this technique with the idea of tracking down the evaporation kinetics of the sensing agent.

## Figures and Tables

**Table 1:** Experimental and theoretical comparison of porosity values for two different dielectric layers.

Theoretical		Experimental	
Porosity (%)	Refractive Index	Porosity (%)	Refractive Index
50	2.06342 − 0.049064i	39%	3.4
66	1.9903 − 0.025293i	74%	1.62

## Supplementary Materials

The following are available online at http://www.mdpi.com/1996-1944/11/6/894/s1, Videos Trial235%.mp4, Trial270%.mp4: Time-resolved spectroscopy of ethanol examples. And informationsupport.docx: The theory of porous silicon microcavity bandgap structure is also available online.
